# Development of the entrustable professional activity ‘medication reconciliation’ for clinical pharmacy

**DOI:** 10.1186/s12909-024-05504-0

**Published:** 2024-05-24

**Authors:** Ula Bozic, Matthias J. Witti, Schmidmaier Ralf, Martin R. Fischer, Jan M. Zottmann, Yvonne M. Pudritz

**Affiliations:** 1 Institute of Medical Education, LMU University Hospital, LMU Munich, Pettenkoferstr. 8a, 80336 Munich, Germany; 2 Doctoral Programme Clinical Pharmacy, LMU University Hospital, LMU Munich, Marchioninstr. 15, 81377 Munich, Germany; 3 Department of Medicine IV, LMU University Hospital, LMU Munich, Ziemsenstr. 5, 80336 Munich, Germany; 4 Pharmacy Department – Clinical Pharmacy and Pharmacotherapy, LMU Munich, Butenandtstr. 5-13, 81377 Munich, Germany; 5 Pharmacy, LMU University Hospital, LMU Munich, Marchioninistr. 15, 81377 Munich, Germany

**Keywords:** Entrustable professional activities, Clinical pharmacy services, Pharmacy education, Assessment

## Abstract

**Background:**

Entrustable professional activities (EPAs) are observable process descriptions of clinical work units. EPAs support learners and tutors in assessment within healthcare settings. For use amongst our pharmacy students as well as pre-registration pharmacists we wanted to develop and validate an EPA for use in a clinical pharmacy setting at LMU University Hospital.

**Methods:**

The development of the clinical pharmacy EPA followed a set pathway. A rapid literature review informed the first draft, an interprofessional consensus group consisting of pharmacists, nurses, and medical doctors refined this draft. The refined version was then validated via online survey utilising clinical pharmacists from Germany.

**Results:**

We designed, refined and validated an EPA regarding medication reconciliation for assessment of pharmacy students and trainees within the pharmacy department at LMU University Hospital in Munich. Along with the EPA description an associated checklist to support the entrustment decision was created. For validation an online survey with 27 clinical pharmacists from all over Germany was conducted. Quality testing with the EQual rubric showed a good EPA quality.

**Conclusions:**

We developed the first clinical pharmacy EPA for use in a German context. Medication reconciliation is a suitable EPA candidate as it describes a clinical activity performed by pharmacists in many clinical settings. The newly developed and validated EPA ‘Medication Reconciliation’ will be used to assess pharmacy students and trainees.

**Supplementary Information:**

The online version contains supplementary material available at 10.1186/s12909-024-05504-0.

## Background

Entrustable professional activities (EPAs) are observable process descriptions of clinical work units consisting of knowledge, skills and attitudes [1]. These descriptions can vary in scope and range from small, discrete, tasks like taking a patient’s temperature, to complex tasks like discharge planning. The concept of EPA within healthcare education was introduced in [2] in response to the shift in medical education with a focus on competency-based medical education. There are currently only a few objective assessment tools to assess clinical competencies. Competencies are commonly used by accreditation bodies albeit often only considering what is desirable and not necessarily observable [3]. EPAs are a good tool to close this gap between theoretical knowledge and the transfer into clinical practice. In this regard, they can be assigned three distinct roles: (1) they can help learners understand what is expected of them in performing a particular activity by describing in detail, the tasks and subtasks to be performed within a unit of professional practice; (2) they can be used for structuring curricula of healthcare professions; (3) they can be utilized as assessment tools to evaluate the clinical performance of the learner.

EPAs have attracted interest worldwide in healthcare education since their introduction in 2005 and are currently used in Germany, Switzerland, Canada and United States. In these countries, EPAs help to structure curricula for medical education and/or postgraduate medical training [4–7]. In pharmacy education, EPAs structure the specialization for community pharmacist in postgraduate education in the Netherlands [8]. In Australia, EPAs are used as workplace-based assessment tools to support postgraduate training in pharmacy [9]. In the Doctor of Pharmacy (PharmD) education in the United States, EPAs are linked to guidance documents [10]. EPAs are highly context-specific and, therefore, not easily transferable between settings [11, 12]. For example, standards or guidelines might differ between hospitals or settings. Tailoring EPAs to local conditions ensure their applicability and effectiveness, leading to a more meaningful and accurate assessments of clinical performance. However, EPAs applicable to multiple professions are not a feasible construct [13]. By default, EPAs are designed to be entrusted to an individual. Furthermore, EPAs constructed to measure in and accurate assessment of clinical performance. Although the concept of transdisciplinary EPAs, which are applicable in multiple disciplines or specialties, has recently been introduced, i.e. the EPA can be used in both professions but the assessment will be performed individually [12, 13]. Furthermore, EPAs constructed to assess interprofessional collaboration across different health professions [14] do not align with the features of EPAs described in literature [13]. While some clinical activities are interprofessional by nature and cannot be carried out in any other way, the situation is different for other activities. Ward rounds, for instance, can involve doctors only, but nurses and pharmacists might also participate. Therefore, the competencies required for interprofessional collaboration (and their level) that a trainee should have to carry out this individual activity should be specified in every EPA.

An important feature of EPAs is the assessment based on the EPA description and successive entrustment decision. One possibility to link EPA assessment to an entrustment decision is the “Level of Supervision (LoS)” scale consisting of five levels [15]. The first level corresponds to a novice learner who is primarily an observer and not authorized to actively engage in practice. Levels two and three indicate increasing degrees of autonomy, yet still requiring supervision from more experienced clinicians. At stage four, the trainee can work independently with distant supervision. Finally, level five signifies the attainment of advanced competence, where the individual is capable of supervising and guiding other clinicians. This hierarchical scale allows for a systematic assessment of the trainee’s progression and proficiency within the specific clinical context.

In German-speaking countries interprofessional training wards have just begun to include pharmacy students. We believe that EPAs developed specifically for clinical pharmacy can help to address the need for objective assessment tools for clinical competence of pharmacy students in this region.

Three suitable candidates were identified by the task group of pharmacists for a pharmaceutical EPA: medication reconciliation; patient record review; discharge management. Each of these activities are independent of each other, have a defined beginning and end, and can be entrusted to pharmacy students. As medication reconciliation is a significant part within the daily routine of clinical pharmacists, this activity was eventually selected for EPA development.

The aim of this study was to develop and validate a pharmaceutical EPA as an objective assessment tool including a corresponding checklist for use within an interprofessional training ward in Munich, Germany.

## Methods

**Fig. 1:**
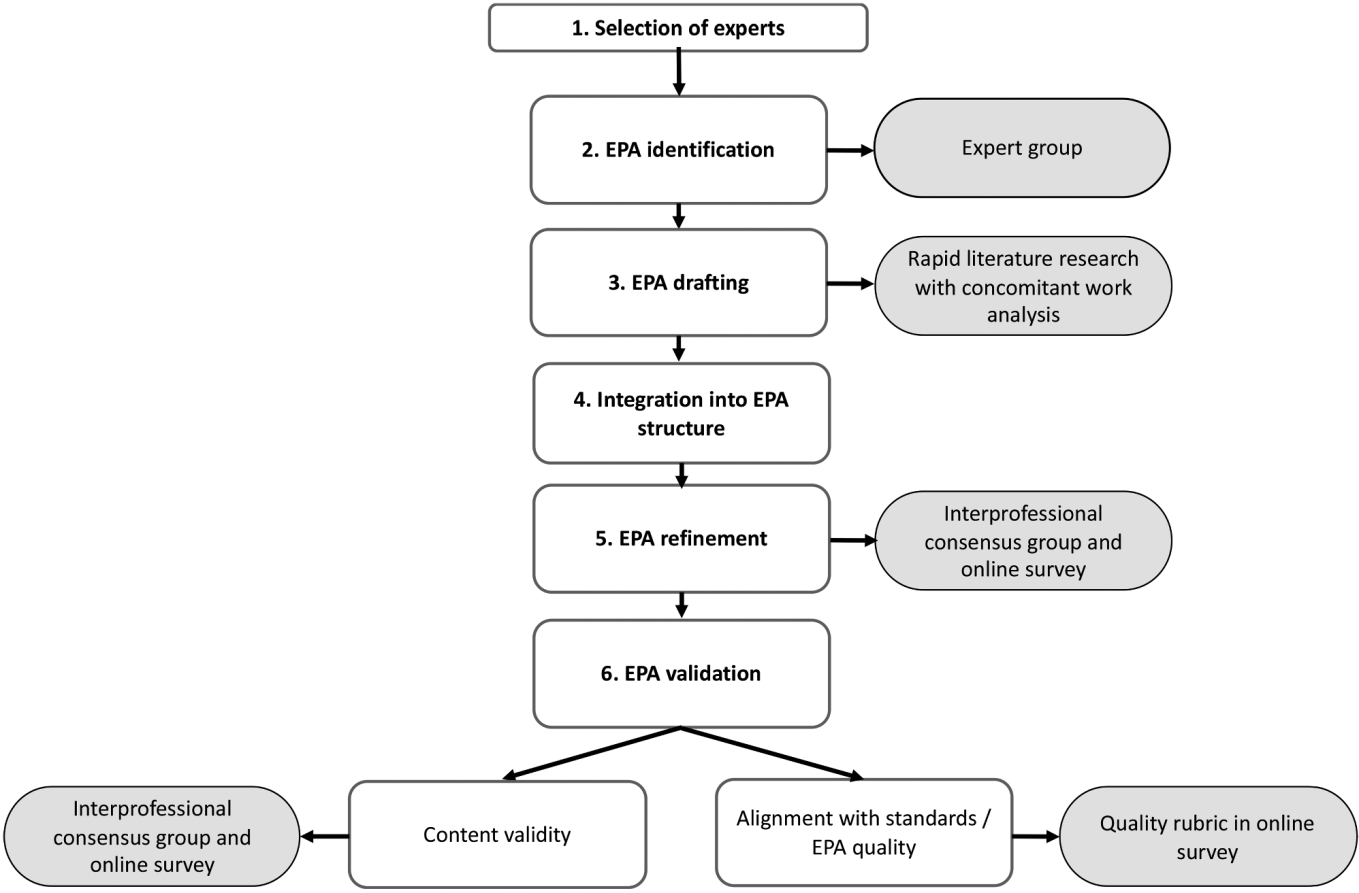
Utilised development pathway for EPA development [16]

For the EPA development, we followed a pathway with several distinct steps as depicted in Fig. 1. This included (1) selection of experts, (2) identification of suitable clinical activities, (3) a rapid literature research with concomitant work analysis, (4) integration of findings into the EPA structure as proposed by Ten Cate [1], (5) EPA refinement and (6) validation/evaluation, both with an interprofessional consensus group and online survey.

### Selection of experts

Expertise from two specialist areas was required for the development of the EPA - including clinical pharmacy expertise, with real-life experience in a ward-based setting, and on the other hand, methodology knowledge concerning EPAs. As the EPA was intended for use on an interprofessional training ward, we also wanted interprofessional involvement from medical doctors and nurses [17, 18]. With the aim of obtaining a representative sample of experts, we decided to include clinical pharmacists from rural and urban hospitals across Germany to incorporate their unique views on clinical activities.

### Rapid literature review and concomitant work analysis

Existing local standard operating procedures and guidelines for the selected activity were chosen and supplemented by a rapid literature search [19], that enabled a timely identification of the main literature on the topic. In addition, work analysis by the clinical pharmacists at LMU University Hospital identified authentic activities and sub-activities required for the selected EPA candidate.

### Integration of findings into EPA structure

We followed a previously described EPA structure [1] and used the results of the rapid literature review and work analysis to fill each of the eight sections (as shown in Table 1).

**Table 1:** Common EPA structure as described by Ten Cate [1]

Section	Description
1	Title
2	Specification and limitations
3	Potential risks in case of failure
4	Most relevant competency domains
5	Required knowledge, skills, attitudes, and experiences to allow for summative assessment
6	Information sources to assess progress and support summative entrustment
7	Entrustment/Supervision level expected for training level
8	Time period to expiration if not practiced

### EPA refinement & validation

EPA refinement took place in two distinct steps. First, an interprofessional consensus group discussed the proposed EPA. Focus was the compliance with EPA methodology. The interprofessional consensus group consisted of seven experts from the LMU University Hospital. Members of this panel had methodological knowledge of EPA development or brought practical clinical experience of the topic to the table. Experts were made up of physicians (1), nurses (3), clinical pharmacists (2), and a pedagogical expert. The interprofessional consensus group met online. The topic guide for the group discussion was based on the EQual rubric [20]. The questions aimed at completeness and comprehensibility of the proposed EPA. The meeting was moderated and with the consent of all participants recorded to aid analysis. In addition, comments were annotated live during the meeting on the proposed EPA and EPA checklist. The version of EPA that was completed at the end of the group discussion was then distributed for validation via an online survey.

For clinical and external validation, an online survey with clinical pharmacists followed. Eligible were clinical pharmacists working in a German hospital. Invitations to take part in the survey were sent out via the mailing list of hospital pharmacists in Germany, with more than 2500 members (*Arbeitsgemeinschaft Deutscher Krankenhaus-Apotheker* – ADKA). The survey was structured in three sections: (1) refinement and validation of EPA content, (2) EPA quality, (3) demographics. The full survey is available on request.

For the first section, refinement and validation of EPA content, the clinical pharmacists were asked to evaluate for completeness of individual EPA sections and express their opinion regarding the use of EPA as an assessment tool in practice. For evaluation of EPA quality (Sect. 2 of the survey) we used the EQual rubric by Taylor et al. [20], which has shown to measure EPA quality reliably and has been recommended by multiple authors [1, 17, 21]. EQual is a criterion-based tool that uses descriptive anchors in each of its items. The original rubric consists of 14 items. For our project, we worked with a slightly modified version with twelve items, which were divided into three subscales: Items 1–5 include statements about EPA as a discrete activity, focusing mainly on the external structure of the EPA, e.g., “This EPA has a clearly defined beginning and end”. Items 6–9 refer to EPA as entrustable, essential and important task of the profession. Finally, items 10–12 aim to evaluate EPA as educational tool and importance for the trainees. All items are shown in Table 2. The rubric was translated from English to German following the FACIT (Functional Assessment of Chronic Illness Therapy) translation methodology [22, 23]. Socio-demographic variables e.g., sex, age, profession, additional training, years of experience were asked in Sect. 3 of the survey.

**Table 2:** EQual Rubric Items, sorted in three clusters [20]. Excluded items are at the bottom

Items from the original EQual rubric	
Nr	Item
**Discrete activity**	
1	This EPA has a clearly defined beginning and end.
2	This EPA is independently executable to achieve a defined clinical outcome.
3	This EPA is specific and focused.
4	This EPA is observable in process.
5	This EPA is measurable in outcome.
**Entrustable, essential, and important task of the profession**	
6	This EPA describes work that is essential and important to the profession.
7	Performing this EPA leads to recognized output or outcome of labour.
8	The performance of this EPA in clinical practice is restricted to qualified personnel.
9	This EPA addresses professional work that is suitable for entrustment.
10	This EPA requires the application of knowledge, skills, and/or attitudes (KSAs) acquired through training.
11	This EPA involves application and integration of multiple domains of competence.
12	This EPA describes a task and avoids adjectives (or adverbs) that refer to proficiency.
**Excluded items from original rubric**	
13	This EPA is clearly distinguished from other EPAs in the framework.
14	The EPA title describes a task, not qualities or competencies of a learner.

### EPA checklist for assessment

Following the example of Wölfel et al. [24], an EPA checklist was created based on the EPA description by two clinical pharmacists. The checklist was then tested with a group of foundation pharmacists at our hospital (*n* = 7). Inter-rater reliability (for two evaluators) was calculated across all items representing individual activities for ten observations.

### Statistical analyses

For statistical analysis of the survey the data was exported into Excel (16.69.1) for calculation of item content validity index (I-CVI), universal agreement score for each item (UA), average of I-CVI scores across items (S-CVI/Ave) and average UA scores across items (S-CVI/UA) [25]. Cut-off point for acceptable CVI was set at a minimum of 0.78 [26]. EPA quality was analysed with a slightly modified version of the EQual rubric [20]. Items of this rubric were rated on a scale from 1 to 5. In addition to the three sub-scales an average for the whole scale (12 items in total) was calculated. A cut-off score of 4.07 or more was set as an indicator for good EPA quality.

### Ethics

Ethics approval was granted by the ethics committee at LMU University Hospital (approved project number 20–0797).

## Results

### Selection of experts

Of those invited to participate in the online survey via the ADKA mailing list, 34 clinical pharmacists ultimately took part, seven of whom had to be excluded due to missing data. While the total number of experts included in the calculations was only 27, all federal states of Germany were represented.

### Rapid literature review and concomitant work analysis

We employed a mixed-methods approach to identify relevant papers and tasks for medication reconciliation. We searched Pubmed, Google Scholar and the archives of *Krankenhauspharmazie* (official journal of the German Association of Hospital pharmacists) for publications on medication reconciliation especially regarding the process description in German or English language published between 2011 and 2021. An additional search was conducted in the online catalogue of the LMU University Library (OPAC). A total of 14 references was included in the rapid literature review [27–40] We also included the existing standard operating procedure (SOP) ‘Medication Reconciliation (MedRec)’ from the hospital pharmacy at LMU University Hospital. A group discussion between three clinical pharmacists with experience in the medication reconciliation process at the LMU University Hospital identified the relevant steps in the respective processes to develop a chronological order of tasks required.

### Integration of findings into EPA structure

Ten Cate described eight sections as part of an EPA as shown in Table 1. The LMU-specific SOP MedRec and the Best Possible Medication History (BPMH [27]), as described by the WHO were the basis for the content development of the EPA. To populate Sect. 5 of the EPA structure, catalogues of learning objective from Germany (NKLM) and Switzerland were used [35, 36]. We also consulted guidelines on good pharmacy practice by WHO [37] for defining appropriate attitudes. Section 4 was filled with applicable framework analogous to CanMEDS roles in medicine [39]. The ISMP List of High-Alert Medications helped to specify limitations in Sect. 2 [38]. Our work analysis supplemented Sects. 2 and 5.

### EPA refinement & validation

Item content validity index was calculated and was ranging from 0.81 to 0.89 for each individual question S-CVI/Ave was 0.87 and S-CVI/UA 0.00, as no universal agreement was reached. Nevertheless, the calculated values have exceeded the set goal of at least 0.78 as shown in Table 3.

**Table 3:** Calculated content validity indices for EPA ‘Medication Reconciliation’. Questions were translated from German

Question	Experts in Agreement	I-CVI	UA (universal agreement)
1 The present EPA comprises the essential activities and content descriptions of the medication history for me.	24	0.89	0
2 For me, this EPA covers all the important knowledge that the learner should have in order to be able to take a medication history.	22	0.81	0
3 For me, this EPA covers all the important skills that the learner should have in order to be able to take a medication history.	24	0.89	0
4 For me, this EPA covers all the important behaviour that the learner should have in order to be able to take a medication history.	24	0.89	0
	**S-CVI/Ave**	**0.87**	
	**S-CVI/UA**	**0.00**	

When asked for their preferred method of assessment, 62.96% of the survey participants chose direct and focused observation using an associated checklist. The assessment of the finalized medication plan based on the performed medication reconciliation was favoured by 22.22%. Least supported was a feedback discussion (14.81%). Regarding the frequency of EPA assessment, survey participants were divided: 51.85% opted for a repeated evaluation of 4–6 times to reach a verdict on learners’ capability to practice this activity, whereas 48.15% viewed 2–3 times as acceptable. Against this backdrop, we decided to include an assessment for at least 4 times in our EPA description.

Participants were asked to vote for expected LoS depending on stage of training for undergraduate students, foundation pharmacists, and licensed pharmacists (as shown in Table 4). Thus, we decided that LoS 1 and 2 can be expected for undergraduate students, LoS 2–3 for foundation pharmacists. Licensed pharmacists should perform this EPA at LoS 3–5 depending on their experience.

**Table 4:** Results for acceptable Level of Supervision (LoS) depending on experience. Survey participants (*n* = 27) could choose more than one suitable LoS

LoS			
	Undergraduate student	Foundation pharmacists	Licensed pharmacist
1 ‘No permission to act’	70.37%	11.11%	7.41%
2 Permission to act with direct supervision, pro-active supervision present in the room’	66.67%	48.15%	22.22%
3 ‘Permission to act with indirect supervision, not present but quickly available if needed’	7.41%	85.19%	74.07%
4 ‘Permission to act under distant supervision (not directly available, “unsuoervised”)’	0	29.63%	88.89%
5 ‘Permission to provide supervision to junior trainees’	9%	0	29.63%

For the ‘expiry date’, i.e., how often the EPA summative assessment should be repeated, 59.26% of the survey participants chose every two years. When asked if they would consider using this EPA as an assessment tool as part of their everyday clinical life, 77.78% agreed. The relevant EPA sections were revised accordingly to these results (final version available as supplementary data).

Table 5 shows the results for the EQual rubric. The overall mean score was 4.22 (SD 0.87), indicating a good quality.

**Table 5:** Results of EPA quality calculations on the basis of EQual tool for EPA Medication Reconciliation. SD – standard deviation; SEM – standard error of the mean

Item	Mean		SD	Item		Mean	SD	SEM
1 This EPA has a clearly defined beginning and end.	4.6		1.0	1–5	Discrete activity	4.16	0.97	0.084
2 This EPA is independently executable to achieve a defined clinical outcome.	3.5		1.1					
3 This EPA is specific and focused.	4.4		1.0					
4 This EPA is observable in process.	4.4		0.8					
5 This EPA is measurable in outcome.	3.9		0.7					
6 This EPA describes work that is essential and important to the profession.	4.1		0.9	6–9	Entrustable, essential, and important task of the profession	4.32	0.81	0.078
7 Performing this EPA leads to recognized output or outcome of labour.	4.4		0.7					
8 The performance of this EPA in clinical practice is restricted to qualified personnel.	4.1		0.8					
9 This EPA addresses professional work that is suitable for entrustment.	4.7		0.7					
10 This EPA requires the application of knowledge, skills, and/or attitudes (KSAs) acquired through training.	3.7		0.9	10–12	EPA as Educational Tool	4.17	0.77	0.086
11 This EPA involves application and integration of multiple domains of competence.	4.5		0.6					
12 This EPA describes a task and avoids adjectives (or adverbs) that refer to proficiency.	4.3		0.6					
				**SD**	**SEM**			
Overall Score		**4.22**		**0.87**	**0.048**			
Score range		**3.3–4.8**						

### EPA Checklist

The final EPA checklist (supplementary data) grouped observable activities in three major categories: (1) patient interview; (2) preparation of the medication plan and documentation; (3) attitude. Each observable category included several activities and was rated on a scale level of supervision 1–5. The option ‘not applicable’ can be chosen if an activity is not observed. Ten observations of foundation pharmacists performing a medication reconciliation during their daily work were evaluated by two clinical pharmacists as independent raters and the overall Cohen`s Kappa was calculated to be 0.83, indicating an almost perfect agreement [41]. The use of this checklist allowed for a structural assessment that provided insight into the trainee’s current level of performance. The average score derived from the checklist provides a valuable indication of the trainee’s level of progress along the learning continuum.

## Discussion

The process of medication reconciliation on hospital admission was identified as a suitable clinical activity for an EPA in clinical pharmacy. Following a described development pathway, we succeeded in developing and validating EPA Medication Reconciliation for pharmacists and pharmacy students within a German context.

As we could base the EPA description on the local SOP, a rapid literature review quickly and effectively led to an elaborated and detailed EPA draft, eliminating the need for a time- and resource-consuming Delphi survey as favoured by others [17, 18, 42]. Since there is yet no standardized published approach to develop EPAs for a clinical pharmacy context, we developed a pathway proposal [43] and applied this to the development of this EPA. The interprofessional consensus group proved to be very helpful in providing the content for potential risks to patients, suitable CanMEDS domains which could be adapted to a clinical pharmaceutical context, e.g., ‘pharmaceutical expert’, ‘communicator’, as well as the section on expected knowledge, skills, and attitudes. Interprofessional points of view were helpful to provide a wholesome and complete EPA description. The advantage of utilizing such a consensus group discussion was the timely resolve of any issues. Choosing local experts, i.e., clinicians familiar with the procedures within the clinic, also allowed for a ‘personalised’, hospital-specific EPA description. Again, within the usual workload and limited time of healthcare professionals the consensus group allowed for a fast and time-efficient development of the EPA description and checklist. Many activities in clinical pharmacy are based on guidelines and SOPs, thus supporting first drafts of EPA description. For refinement purposes it is necessary to involve local experts of the chosen clinical activity. In our case, the local experts added more information with respect to the required specification and the limitations of medication reconciliation.

In both, the interprofessional consensus group as well as the online survey group, there was a discussion about the entrustment decision and the consequent definition of LoS. As we could identify different target participants for the EPA, e.g. students, nurses, foundation pharmacist and fully licensed pharmacists we decided to provide two solutions for the entrustment decision, one ad hoc, one summative, as shown in the EPA description (shown in supplementary data). The ad hoc entrustment decision allows for a quick assessment with only a few sources of information, *e.g*., only one observation with application of the checklist or review of the medication plan. These different types of assessment allow for distinction between the different target participants. The ad hoc assessment would be feasible for assessment of an experienced clinical pharmacist returning to medication reconciliation. The summative entrustment decision however might be more suitable for students and foundation pharmacists as it is based upon a long-term assessment with several sources of information. The EPA is applicable to a wide range of hospital wards and patients. Limitations mentioned in the EPA description identify patient types that require closer supervision by a pharmacist to ensure patient safety throughout the process. EPAs can be useful as a feedback instrument. Learners, i.e. students and trainees, can gauge expectations of them before assessment in terms of knowledge, skills and behaviours. By performing an assessment based on an EPA the checklist can help the learner and assessor alike to identify areas that still require gaps in learning. This can in turn inform and support their further professional development plan. Another use of EPA could be within a multi-source feedback or appraisal talk depending on the relevant trust’ or employer’s structures.

Even though in the literature EPAs are typically described as very specific to a single clinical context [10], we believe EPAs can be transferred to different settings to a certain degree. Modifications might be necessary e.g., in the specification part of EPA, as the chronological process of clinical activities is depicted there, which might differ between clinical specialties or hospitals. As we could observe in our EPA development process, the health care professions could also differ for specific clinical activities. For instance, at the LMU University Hospital medication reconciliation is performed by clinical pharmacists supported by pre-registration trainees - other hospitals in Germany involve pharmacy technicians in this process. This was reflected in the request of some online survey participants to include pharmacy technicians in the EPA description, underlining the importance of adjusting EPAs to local settings. Since development of an EPA is a lengthy process, modifications seem like a practical solution in wanting to transfer EPA to other contexts.

### Strengths & limitations

The response rate to the online survey was rather low. However, we were still able to recruit a variety of clinical pharmacists from all over Germany with a variety of backgrounds and years of experience. All survey participants were experienced in medication reconciliation in varying degrees. This ensured that we included a multitude of views. A common approach in EPA development for refinement and validation is the Delphi approach. Due to time constraints, we opted for an online survey instead and were able to develop an EPA with satisfactory quality. Further studies on this may be necessary, but online surveys could be a time-efficient alternative to the Delphi approach for the development of EPAs in the pharmacy context (and possible beyond).

Following a set pathway was useful to create a well-rounded EPA description. Using a mixed methods approach, combining a rapid literature review, input from different healthcare professionals (within the interprofessional consensus group) and from different locations ensured a fast and thorough EPA development.

## Conclusion

To the best of our knowledge, this paper presents the first EPA that describes a clinical pharmacy activity in a German context. The EPA Medication Reconciliation is currently being used as an assessment tool within different clinical contexts at LMU Munich. A field validation using an ethnographic qualitative design analogous to Schmelter et al. [43] at an interprofessional training ward could eliminate any remaining doubts about its applicability.

In the future, more EPAs should be developed for clinical pharmacy profession for German context, following the example from medical education. As the number of clinical pharmacists increases and the range of clinical pharmacy services provided expands, the competencies of clinical pharmacists require assessment. EPAs appear to be suitable tools for the task. An easy-to-follow, time-efficient and standardized approach to EPA development should be established to enable more EPAs to be developed that meet the standards proposed in the literature.

### Electronic supplementary material

Below is the link to the electronic supplementary material.

Supplementary Material 1

Supplementary Material 2

## Data Availability

The datasets used and/or analysed during the current study are available from the corresponding author on reasonable request.

## Abbreviations

BPMH: best possible medication history
CanMEDS: Canadian Physician Competency Framework
EPA: entrustable professional activities
I: CVI–item content validity index
ISMP: Institute for Safe Medication Practices
LMU: Ludwig–Maximilians–Universität
LoS: Level of Supervision
SD: standard deviation: SEM–standard error of the mean
SOP: standard operating procedure
UA: universal agreement
WHO: World Health Organization
