# Supplementary material for: Development of the entrustable professional activity ‘medication reconciliation’ for clinical pharmacy
Source: BMC Med Educ. 2024 May 24;24:568. doi: 10.1186/s12909-024-05504-0 (PMC11127371; doi:10.1186/s12909-024-05504-0)
Supplement: Supplementary file 1 — Supplementary Material 1 [file 12909_2024_5504_MOESM1_ESM.pdf]

Trainee name: Date:

Checklist EPA Medication Reconciliation (MedRec)

Areas of competence	Observable tasks	n.a.	Required Level of Supervision 1	2	3	4	5
**Medication Reconciliation patient conversation**	• Before the interview: collect as much information (e.g., medication schedules, doctor's letters, previous medical history) about the patient as appropriate for assessment.						
	• Short introduction of own's role to patients and/or relatives.						
	• Confirms patient's identity (first and last name, date of birth)						
	• Conducts medication reconciliation in line with the data collection sheet<br>☐ Height & weight ☐ Past medical history ☐ Regular medication (Inc. dose, strength, dosage form, if necessary indication, i.e. antibiotics, OAKs) ☐ as required meds ☐ recently discontinued meds ☐ OTC preparations ☐ special dosage forms/application systems (e.g. inhalators, eye drops, NMWH) ☐ Known drug allergies ☐ Application problems						
	• If patient is unable to provide information about his/her medication (lack of knowledge or limited cognitive abilities), the learner inquires about the patient's medication via alternative sources of information, e.g., relatives, GP ☐ Nr of information sources used:						
**Preparation of medication plan & documentation**	• Performs a medication analysis type 2b/3 according to the guideline (medication, clinical data, if possible, conversation with patient) and documents any medication related problem.<br>☐ strength & dosing interval ☐ interactions ☐ contraindications ☐ duplicate medication ☐ length of treatment<br>☐ renal function ☐ untreated medical conditions/ medication without indication ☐ Adherence to guidelines<br>☐ pre-operative discontinuation (surgical patients only)						
	• Switches patents' own medication to suitable alternatives according to the local formulary; establishes any need for patient-specific orders (e.g., non-formulary, cytotoxics, special order items).						
	• Passes on the medication plan (in writing or electronically) to the responsible ward team (doctors + nurses).						
	• Documents their work according to the local SOP (e.g., patient's name, date of birth, date of MedRec, ward, number of medications, time spent).						
**Behaviour**	• Takes a proactive stance, recognises, and responds to need for action.						
	• Demonstrates consistent, predictable, and conscientious behaviour.						
	• Communicates in a structured and respectful way with patients and, if applicable, with relatives; maintains eye contact; avoids technical terms; if possible, asks open questions; protects patient's privacy; decides in the best interest of the patient; is understanding of patients' needs and emotions; remains calm and polite towards patient even in conflict situations.						
	• Works well with others; performs drug history within a reasonable time frame and maintains an overall view of the clinical situation.						
	• Recognises his/her own limitations and possibilities and asks for support when necessary/required.						

Level of supervision (LOS):

1 trainee is only present to observe, not to enact an EPA
2 direct, pro-active supervision
3 trainee is allowed to carry out the EPA with indirect, reactive, supervision.
4 trainee is allowed to work unsupervised („distant supervision")
5 trainee is able to supervise junior trainees

Trainee name: Date:

Entrustment Based Discussion:

1. Does the trainee know what must be done resp. what have they done? (case explanation/presentation)

2. Does the trainee demonstrate sufficient background knowledge? (Clinical decision making, rationale, indication, pathophysiology).

3. Is the trainee aware of the risks and possible complications of the activity? (How did the trainee manage risks?)

4. How would the trainee handle unusual patients/ rare findings/ rare medications/ emerging risks/ complications?

5. Is the trainee prepared to take on (more) responsibility?

EPA LMU
