# Supplementary material for: Development of the entrustable professional activity ‘medication reconciliation’ for clinical pharmacy
Source: BMC Med Educ. 2024 May 24;24:568. doi: 10.1186/s12909-024-05504-0 (PMC11127371; doi:10.1186/s12909-024-05504-0)
Supplement: Supplementary file 2 — Supplementary Material 2 [file 12909_2024_5504_MOESM2_ESM.docx]

Appendix 1

| **EPA Title** | **Medication Reconciliation (MedRec)** |
| --- | --- |
| **Description (Specification and limitations)** | The trainee (pharmacy student, foundation pharmacist, pharmacist) can independently conduct a discussion about a patient's medication and prepare a medication plan for the hospital stay with instructions for the nursing staff and the doctors, as well as document it accordingly.  **The execution of this EPA includes:**   - Preparation of the patient interview by compiling relevant medication schedules, doctor's letters, and/or previous medical history. - Discuss the patient's medication on admission to hospital using structured questionnaires. If the patient is unable to provide any information himself/herself, conduct the discussion with the family doctor/nursing home/relatives/primary pharmacy. The use of two independent sources of information is also always desirable.   - Medication to be recorded includes:     - Current as well as recently discontinued or newly prescribed,     - Rx and OTC (including food supplements, herbal preparations, homeopathic remedies)     - Regular as well as required medication     - Special dosage forms (e.g., eye drops, inhalers, transdermal systems, cremes, ointments)     - Strength, dosing interval and method of use     - Gender-specific medication (e.g., contraceptive pill)   - During the interview, the following additional questions are asked: problems with taking medication (including non-adherence), weight, height, diagnoses, planned surgery, functional disorders of the kidneys (including the need for dialysis) or the liver, allergies and intolerances and their symptoms, problems with blood clotting, alcohol, nicotine and grapefruit juice consumption, existence of PEG/NG or stoma. - Medication analysis Type 2b/3 (Medication, clinical data, if possible, conversation with patient). - Switch of medication according to the local formulary and establishing any need for patient specific orders (e.g., non-formulary, cytotoxics, special order items) - Preparation of a medication plan with notes on drug-related problems, pre-operative discontinuation of medication and patient-specific orders. - Presentation of patient and prepared plan to the supervising pharmacist and correction by pharmacist if necessary. - Passing on (in writing) the medication plan to the responsible team on the ward (doctors and nursing). - If indicated: taking care of necessary documentation, e.g., name, date of birth, date of interview, ward, number of medications, time spent.   **Limitations:**  The following patient groups require closer supervision by a pharmacist:   - Patients with polypharmacy (i.e., more than 5 drugs) – often multiple co-morbidities. - Patients with high-risk medications (according to ISMP list, e.g., anticoagulants, chemotherapeutic agents, insulins, opioids). - Particularly vulnerable patients (e.g., immunosuppressed patients, patients requiring dialysis, patients with resorption difficulties). - Unstable or critically ill patients. |
| **Potential risks in case of failure** | Treatment of a patient with an incorrect or unsuitable medicine (e.g., strength, dosage, active ingredient) or failure to treat an indication due to lack of knowledge or communication deficits. Improper use of the medicine due to ignorance, lack of information or inability (e.g., sharing tablets) on the part of the patient. As a result, negative consequences are possible, which may be related to the medication administered or missing. In addition to a persistence of treatable diagnoses and symptoms, mistreatment can also lead to patient uncertainty. |
| **Most relevant competency domains (CanMEDS)** | Pharmaceutical Expert, Communicator, Collaborator, Professional |
| **Required knowledge, skills, and behaviour** | **Knowledge:**  Disease patterns (treatment guidelines and algorithms as well as SOPs of common diseases and health disorders), drug groups and substances (approved in Germany), indications, contraindications (due to age, gender, diseases, allergies, organ function), drug selection (evidence), pharmacotherapy in special situations (organ insufficiency, pregnancy, lactation, geriatrics, paediatrics, addiction patients), potentially inadequate medication (PIM), double medication, drug and food interactions, strengths and dosage intervals of medications, timing of medication intake (also in connection with meals), duration of therapy, preoperative discontinuation of medication, side effects of medications (also QTc time prolongation and anticholinergic stress), prescription cascades, medication application/use (e.g., injections, inhalers, inhalers). (e.g., injections, inhalers), suitable dosage forms, laboratory values (relevant for drug therapy), self-medication (especially indications and limits), proper storage of drugs.  **Skills:** Communication:   - Interaction with patients and their relatives   Presentation of one's own role to patients and/or relatives, confirmation of patients' identity, assessment if further sources of information are necessary in addition to the patient interview (assessing patients' cognitive abilities, determining knowledge deficits), ability to obtain information about patients, structured communication within the framework of the interview, shaping of a trusting pharmacist-patient relationship, recognition of typical drug side effects, adherence and application problems.   - Interaction within the (interprofessional) ward team   Understanding one's own role in the team, structured exchange with the doctor and ward staff about the medication plan (e.g. SBAR^[[1]](#footnote-1)^ scheme), goal-oriented communication in a professional team  Conduct during drug history taking (factual, professional)  Preparation and presentation of the medication plan: Switch to formulary drugs, recognition of patient-specific requirements (e.g., non-formulary drugs, cytotoxics, special order drugs). Medication analysis type 2b/3. Effective use of available software or other electronic sources. Ability to file the prepared plan correctly (enter electronically or submit in paper form).  Documentation: e.g., name, date of birth, date of interview, ward, number of medications, time spent.  **Behaviour:** Empowerment: Recognise the need for and perform a drug history even if it is outside the strict definition of one's responsibilities and practice of adaptive competence.  Reliability: Consistent, predictable and conscientious behaviour guided by a sense of accountability and responsibility.  Patient-centredness: Patient-centred communication (e.g., active listening, avoiding technical terms, asking open questions), understanding patients' needs and emotions and protecting their privacy. Expertise is used for the benefit of the patient and all decisions are justified and made in the best interest of the patient.  Professional understanding: Possession of theoretical and practical knowledge acquired during studies/training to perform a drug history at different sites and in different contexts within an appropriate time frame. An adequate understanding and overall view of the clinical situation.  Humility: willingness to ask for help with drug history; accepting support of doctors and nursing staff; and willingness to learn from mistakes, feedback, and the expertise of others.  A list of observable activities can be found in the associated EPA checklist. |
| **Assessment - Entrustment decision** | - **Ad hoc entrustment decision:** Quick assessment with few sources of information (e.g. only one observation with application of the checklist or review of the medication plan). - **Summative entrustment decision**: Long-term assessment with several sources of information and the character of an authorisation - several times (min. 4x) observation, discussion (min. 4x) and assessment of the medication plan (min. 4x).   Possible tools that can be used for the assessment:   \| **Approach** \| **Methods** \| **Instruments** \| \| --- \| --- \| --- \| \| Observation \| Brief and focussed observation \| Observable activities (checklist) \| \| Review of results \| Evaluation of products/outputs \| Finalised medication plan or medication record \| \| Discussion \| Brief feedback discussion \| Trust-based discussion \| |
| **Expectations regarding the achievement of a certain level of independence** | - **Pharmacy student**: only as observer or direct supervision at all times. - **Foundation pharmacist**: direct supervision at all times or moderate (selective direct) supervision at all times. - **Pharmacist:**  moderate supervision at start of that clinical area, independent execution possible after 4 weeks of experience. Supervise other trainees in the performance of the activity after at least 8 weeks of experience. |
| **Time period to expiration if not practiced** | - 2 years - Sooner if change of clinical area |

1. Situation, Background, Assessment, Recommendation – easy to use, structured form of communication that enables information to be transferred accurately between individuals [↑](#footnote-ref-1)
